# Reconstructing genome mixtures from partial adjacencies

**DOI:** 10.1186/1471-2105-13-S19-S9

**Published:** 2012-12-19

**Authors:** Ahmad Mahmoody, Crystal L Kahn, Benjamin J Raphael

**Affiliations:** 1 Department of Computer Science, Brown University, Providence (RI), USA

## Abstract

Many cancer genome sequencing efforts are underway with the goal of identifying the somatic mutations that drive cancer progression. A major difficulty in these studies is that tumors are typically heterogeneous, with individual cells in a tumor having different complements of somatic mutations. However, nearly all DNA sequencing technologies sequence DNA from multiple cells, thus resulting in measurement of mutations from a mixture of genomes. Genome rearrangements are a major class of somatic mutations in many tumors, and the novel adjacencies (i.e. breakpoints) resulting from these rearrangements are readily detected from DNA sequencing reads. However, the assignment of each rearrangement, or adjacency, to an individual cancer genome in the mixture is not known. Moreover, the quantity of DNA sequence reads may be insufficient to measure all rearrangements in all genomes in the tumor. Motivated by this application, we formulate the k-minimum completion problem (*k*-MCP). In this problem, we aim to reconstruct *k *genomes derived from a single reference genome, given partial information about the adjacencies present in the mixture of these genomes. We show that the 1-MCP is solvable in linear time in the cases where: (i) the measured, incomplete genome has a single circular or linear chromosome; (ii) there are no restrictions on the chromosomal content of the measured, incomplete genome. We also show that the *k*-MCP problem, for *k *≥ 3 in general, and the 2-MCP problem with the double-cut-and-join (DCJ) distance are NP-complete, when there are no restriction on the chromosomal structure of the measured, incomplete genome. These results lay the foundation for future algorithmic studies of the *k*-MCP and the application of these algorithms to real cancer sequencing data.

## Introduction

Nearly all current genome sequencing studies sequence the DNA from a population of cells rather than from single cells. This is because present DNA sequencing technologies cannot sequence the DNA in a single cell without bias-inducing DNA amplification steps. In the majority of applications, sequencing such a population of cells is not problematic because the DNA in every cell is nearly identical. However, there are two notable examples: metagenomics (e.g. environmental sequencing or microbiome studies) and cancer sequencing. In the former case, the genomic differences between cells are due to the presence of mixtures of microorganisms in the sample. In the latter case, the genomic differences between cells are due to somatic mutations that accumulate in individual tumor cells during the progression of cancer [1].

In this paper, we formulate the problem of inferring the organization of each genome present in a mixture in the case where: (1) the individual genomes result from an unknown sequence of genome rearrangements from a known (reference) genome; (2) the adjacencies (breakpoints) of the genomes in the mixture are measured. This situation arises in cancer genome studies where somatic structural aberrations (including inversions, translocations, duplications, deletions, or other rearrangements of large pieces of DNA) induce novel adjacencies, also called breakpoints, that join in the cancer genome two noncontiguous nucleotides from the normal genome. In current cancer sequencing projects, these novel adjacencies are determined from alignments of paired-end reads from cancer DNA to the reference human genome [2,3]. However, these approaches generally do not measure all adjacencies present in the tumor. For example, the quantity of DNA sequence reads (coverage) may be insufficient to measure all adjacencies in all genomes in the tumor, particularly adjacencies that are present in a minority of cancer cells. Moreover, alignment of reads to repetitive regions is challenging, particularly for short reads produced by current sequencing technologies, and thus some adjacencies may not be reliably measured.

We formulate the *k*-Minimum Completion Problem (*k*-MCP) of determining the *k *genomes present in a mixture from a set of measured adjacencies that minimize the total distance between the reference genome and the *k *measured (i.e. cancer) genomes. The *k*-MCP is a general problem that encompasses different subproblems that depend on the genomic distance used and the desired chromosomal content of the measured genomes. We show the following results: (1) A linear time algorithm for the 1-MCP in the double cut and join (DCJ) distance [4] when the desired genome has no restrictions on its chromosomal content; (2) A linear time algorithm for the 1-MCP in the DCJ distance when the desired genome has a single circular or linear chromosome; (3) the *k*-MCP is NP-complete for any distance when *k *≥ 3; and (4) the 2-MCP with DCJ distance is NP-complete when the desired genome has no restrictions on its chromosomal content, or when the desired genome has all circular chromosomes.

We emphasize that the *k*-MCP does not model all the issues arising in cancer sequencing: in particular, we restrict attention to copy-neutral structural variants, and ignore single nucleotide mutations, small indels, or other large copy number aberrations. Single nucleotide mutations and small indels can be addressed separately as they do not produce novel adjacencies of the type studied in *k*-MCP. Copy number aberrations are common in cancer, but appropriate handling of these mutations when measured in a heterogeneous mixture introduces an entirely different set of challenges: e.g. a deletion of a genomic segment in half of the cells in the mixture with a duplication of the same segment in the other half of the cells will be difficult to distinguish from no copy number change. Finally, we assume that all measured adjacencies are real, while in fact there are likely to be false positive adjacencies. Extending the model to consider these additional complexities is left for future work.

In following sections, we first provide a precise formulation of the *k*-MCP and describe related work. Then, we provide algorithms and proofs of the complexity of the problem in various cases.

### Definitions and problem statement

In this section we present some preliminary definitions and give the formal definition of *k*-MCP.

**Figure 1:**
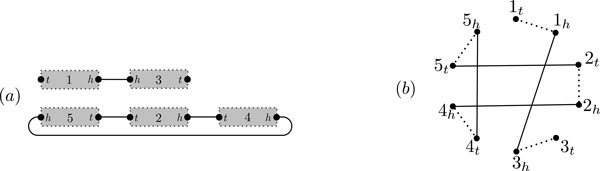
**Genome and genome graph**. (a) A genome  G on the set of genes {1, 2, 3, 4, 5} with two chromosomes (one linear and one circular). $A\left(G\right)=\left\{\left\{1_{h},3_{h}\right\},\left\{5_{t},2_{t}\right\},\left\{2_{h},4_{t}\right\},\left\{4_{h},5_{h}\right\}\right\},T\left(G\right)=\left\{1_{t},3_{t}\right\}$. (b) The genome graph (black edges) of  G with additional edges (dotted) connecting the extremities of the same gene. There is one cycle component and one path component.

A *gene g *is an oriented sequence of nucleotides, with two extremities: a *head g_h _*and a *tail g_t_*. An *adjacency *is an unordered pair of gene extremities. A *genome * G on *n *genes is a set $A\left(G\right)$ of adjacencies such that each of the 2*n *gene extremities in  G is a member of at most one adjacency in $A\left(G\right)$. The gene extremities which are not members of any adjacency in $A\left(G\right)$ are called *telomeres *of  G, and we denote the set of all telomeres by $T\left(G\right)$ (Figure 1-a). Through this work, we assume that the genes of a genome are distinct.

The *genome graph *of a genome  G is a graph whose labeled vertices are the gene extremities in  G, and whose edge set is $A\left(G\right)$. We denote the genome graph of  G by $\text{gr}\left(G\right)$. Because each extremity is in at most one adjacency of $A\left(G\right)$, the graph $\text{gr}\left(G\right)$ is a matching graph (not necessarily perfect). Note that the genome graph is uniquely determined by the genome, and conversely. For convenience, we also define the *augmented genome graph *$\bar{\text{gr}}\left(G\right)$ to be the genome graph augmented with additional edges connecting extremities of the same gene, i.e., $\bar{\text{gr}}\left(G\right)$ is the graph whose labeled vertices are the gene extremities in *G*, and whose edge set is $A\left(G\right)\cup \left\{\left\{g_{h,}g_{t}\right\}|g\;\text{is}\;\text{a}\;\text{gene}\;\text{in}\;G\right\}$.

A *chromosome *of  G is the set of all adjacencies and telomeres of gene extremities in a connected component of the augmented genome graph (Figure 1-b). A chromosome is *linear *(resp. *circular*) if the corresponding connected component is a path (resp. cycle) (Figure 1-b). Note that an adjacency {*g_h_*, *g_t_*} represents a circular chromosome with the single gene g. A genome is *circular *or *linear *if all of its chromosomes are circular or linear, and we say it is *mixed *if it has both circular and linear chromosomes. A genome is *uni*-*chromosomal *if it has only one chromosome, and it is *multi*-*chromosomal*, otherwise. A *chromosomal condition *is a condition on the number or type of chromosomes in a genome. For example we can describe the structure of a genome by two chromosomal conditions: being (i) uni-chromosomal, and (ii) circular.

As described above a paired-end sequencing experiment provides the adjacencies $A\left(G\right)$ of the sequenced genome relative to the genes from a reference genome. However, our knowledge about a genome's adjacencies is typically incomplete. For a set  C of chromosomal conditions, a  C -*partial genome *$G^{\prime }$ on *n *genes is a set of adjacencies $A\left(G^{\prime }\right)$ such that there exists a set $Ā\left(G^{\prime }\right)$ of pairs of gene extremities such that $A\left(G^{\prime }\right)\cup Ā\left(G^{\prime }\right)$ is a genome with chromosomal condition  C. When  C is clear in the context we will say partial-genome instead of  C -partial genome. The problems we study below involve adding the missing adjacencies in  C -partial genomes to complete them into genomes with chromosomal condition  C. Sometimes we have an idea about the number or the structure of chromosomes in a genome. We define a *completion *of a partial genome relative to these chromosomal conditions. If  G is a genome, we say $G^{\prime }\subseteq G$ provided $A\left(G^{\prime }\right)\subseteq A\left(G\right)$. A $completion_{C}$ of a partial genome $G^{\prime }$ is a genome  G with $G^{\prime }\subseteq G$ and satisfying the conditions in  C. When  C is clear in the context, we just say completion instead of ${\text{completion}}_{C}$.

A *multi-genome *is a mixture of genomes with the same set of genes. Formally, the multi-genome  M formed from genomes $G_{1},...,G_{m}$ is a multiset A(M) obtained from $A\left(M\right)=\;{⊔}_{i=1}^{m}A\left(G_{i}\right)$, the disjoint union of $A{\left(G_{i}\right)}^{\prime }s$ (For a multiset S and an element *r*, if *c_S_*(*r*) is the number of copies of *r *in *S*, the disjoint union of two multisets A⊔B is a multiset in which each element *r *appears *c_A_*(*r*) + *c_B_*(*r*) times.). Note that the partition of the adjacencies in A(M) into $A\left(G_{1}\right),...,A\left(G_{m}\right)$ is not known. There is a corresponding *genome graph*, a *multigraph *whose vertices are the gene extremities, and whose edge set is the multiset A(M). We denote the genome graph of a multi-genome  M by gr(M).

The genome graph is related to the *breakpoint graph *in genome rearrangement studies. The breakpoint graph $B\left(G_{1},\ldots ,G_{m}\right)$ of the genomes $G_{1},...,G_{m}$ is an edge-colored multigraph whose labeled vertices are the 2*n *gene extremities and whose edges are all the adjacencies in ${⊔}_{i=1}^{m}A\left(G_{i}\right)$, with each edge assigned a color according to its genome of origin. Thus, the only difference between the breakpoint graph and the genome graph is the lack of edge-coloring in the latter, reflecting our inability to measure the origin of each adjacency.

Our knowledge about a multi-genome can be incomplete. For example a tumor is a mixture of different cancer genomes, and during sequencing process, we obtain a *mixture *of adjacencies from these genomes. We represent the mixtures of adjacencies by a *partial multi-genome*. A partial multi-genome is a multi-set ${⊔}_{i=1}^{m}A\left(G_{i}^{\prime }\right)$, where each $G_{i}^{\prime }$ is partial genome. We define the *genome graph *of a partial multi-genome analogously to a multi-genome.

If *k *is a positive integer and  M is a partial multi-genome, a *k-completion *of  M is a family of *k *genomes $M^{k}=\left\{G_{1},...,G_{k}\right\}$, such that $M\subseteq \;{⊔}_{i=1}^{k}G_{i}$. Note that *existence *of a completion for a partial (multi-) genome is dependent on the structure of the partial (multi-) genome and the chromosomal conditions. Also, the existence of a completion does not imply its uniqueness.

We use a distance function to distinguish between different completions. A *distance *function on pairs of genomes (with the same set of genes), is a measure of dissimilarity between the genomes. Having selected a pairwise distance function we must define a distance between the *k *genomes in a mixture. Motivated by the fact that the different cancer genomes in a tumor are obtained by somatic genome rearrangements from a healthy genome, we model the evolution of the cancer genomes by a rooted tree in which all the cancer genomes are descendants of the healthy one. Suppose  A represents a healthy genome, and $M^{k}$ a mixture of *k *cancer genomes obtained by rearrangements of the genome  A. A *mixture tree *$T_{M^{k},A}$ is a rooted tree on $M^{k}\cup \left\{A\right\},$ such that the root vertex is  A and *k *genomes in $M^{k}$ are (some of) the vertices in $T_{M^{k},A}$. If *ϕ *is a distance function on a pair of genomes, then the *ϕ*-value of $T_{M^{k},A}$, denoted by $\phi \left(T_{M^{k},A}\right)$ is defined as follows:

$$\phi \left(T_{M^{k},A}\right)=\underset{\left\{u,v\right\}\in E}{\sum }\phi \left(u,v\right),$$

where *E *is the set of edges in $T_{M^{k},A}$.

We now define the *k*-Minimum Completion Problem.

*k***-Minimum Completion Problem (***k***-MCP) **Given a  C -partial multi-genome  M, a positive integer *k*, a reference genome  A, and a distance function *ϕ*, find a *k*-completion $M^{k}$ and a mixture tree $T_{M^{k},A}$ such that $\phi \left(T_{M^{k},A}\right)$ is minimum over all *k*-completions and mixture trees. If no *k*-completion exists for  M, we say that this *k*-MCP does not have a *valid *solution. We say the *k*-MCP is *unrestricted *if C=∅, and is *restricted*, otherwise.

As written, the *k*-MCP is a general problem that encompasses many subproblems depending on chromosomal condition set  C and the distance *ϕ*. Common distances in genome rearrangement studies include the *breakpoint distance *[5], the *Hannenhalli-Pevzner *distance [6] (which generalizes the *reversal distance *[7]), and the *double-cut-and-join (DCJ) distance *[4]. Below we will use the DCJ distance, which approximates the other distances [8].

For two genomes $G_{1}$ and $G_{2}$ on the same set of *n *genes, their *double-cut-and-join (DCJ) *distance, denoted by $d_{DCJ}\left(G_{1},G_{2}\right)$, is equal to

$$n-c\left(G_{1},G_{2}\right)-\frac{p\left(G_{1},G_{2}\right)}{2}$$

where $c\left(G_{1},G_{2}\right)$ is the number of cycles in $B=B\left(G_{1},G_{2}\right)$ and $p\left(G_{1},G_{2}\right)$ is the number of paths in *B *with odd number of vertices [8].

*Remark*. When at least one of the ${G_{i}}^{\prime }s$ are circular we have $p\left(G_{1},G_{2}\right)=0$ and *d_DCJ _*(*G*_1_, *G*_2_) = *n - c*. Thus, having a larger number of cycles in their breakpoint graph is equivalent to having a smaller distance.

### Related work

In comparison to other genome rearrangement problems considered in the literature, the *k*-MCP has three distinguishing features. (1) The input is a mixture of adjacencies from multiple genomes and the genome of origin of each adjacency is unknown. (2) The set of adjacencies is incomplete: not every adjacency from every genome in the mixture is measured. (3) The ancestral relationships between the genomes in the mixture are unknown, and might include both "ancestral" and "present day" genomes. Some of these features have been considered individually in other work, but to our knowledge no previous work has considered all three together. The first feature bears some resemblance to the genome halving problem [9] of finding the doubled ancestor genome by minimizing a rearrangement distance. This problem and further generalizations to polyploidization [10] involves partitioning (or coloring) adjacencies to minimize a rearrangement distance. However, in general no adjacencies are missing and the distance is pairwise (i.e., no tree) in contrast to the 2-MCP.

Regarding the second feature, several authors have considered the problem of inferring missing adjacencies in a manner that optimizes a genome rearrangement distance. Notably, [11] and [12] consider the problem of computing reversal distance between pairs of partially assembled genomes that are provided as unordered sequences of contigs. These problems were motivated by limitations in DNA sequence technologies that result in most whole-genome assemblies being highly fragmented and comprised of contigs whose relative ordering is unknown. These problems are variations of the 1-MCP, where the reference genome  A also has missing adjacencies. In particular, [12] orient sets of contigs from two genomes in such a way that the number of cycles in the breakpoint graph of the resulting genomes is maximized, which they note "has been shown to approximate very well the reversal distance between them." However, there is no work on extending this analysis to more than two genomes.

Regarding the third feature, the genome median problem considers the problem of finding an ancestral genome that minimizes the distance between three given genomes [5,13]. This is different from *k*-MCP in that the three individual genomes are known (rather than mixed) and the genomes are complete with no missing adjacencies. Also, in the median problem the topology of the phylogenetic tree has been already inferred, while in *k*-MCP we have to find an optimal topology for the phylogenetic tree as well.

## Results

In this section we first consider the 1-MCP problem. We present linear time algorithms that solve 1-MCP in the cases where: (i) the measured, incomplete genome has a single circular or linear chromosome; (ii) there are no restrictions on the chromosomal content of the measured, incomplete genome.

Next we prove that the unrestricted *k*-MCP is NP-complete when *k ≥ *3 for any distance function *ϕ*. Finally, we show that the unrestricted 2-MCP, and the restricted 2-MCP where all chromosomes are circular (i.e., C={circular}), are NP-complete for DCJ distance.

### 1-MCP

Here, we consider the unrestricted 1-MCP and two restricted versions of 1-MCP problem: (1) the chromosomal condition set is {*circular*, uni-chromosomal}, which we denote by 1-MCP*_c_*; (2) the chromosomal condition set is {*linear*, uni-chromosomal}, which we denote by 1-MCP_ℓ_. We first show that unrestricted version is linearly tractable. Then, we show that we can solve the 1-MCP_*c *_in linear time. Finally, we prove a relation between 1-MCP*_c _*and, 1-MCP_ℓ _which implies that 1-MCP_ℓ _is also solvable in linear time.

Note that 1-MCP_ℓ _is a variation of the Block Ordering Problem (BOP) considered in [12]. In our terminology, the BOP considers two partial genomes, and aims to complete both partial genomes into linear, unichromosomal genomes such that the pairwise distance between the completed genome is minimal. In [12], Gaul and Blanchette provide a linear algorithm for BOP. The algorithm we present for 1-MCP_ℓ _is simpler than the algorithm for the BOP in [12], and our algorithm is obtained from a straightforward algorithm (Algorithm 1 below) which solves 1-MCP*_c _*in linear time.

#### 

We begin with the unrestricted 1-MCP, where we have the following result.

*Theorem *1. The unrestricted 1-MCP with DCJ distance is linearly tractable.

**Figure 2:**
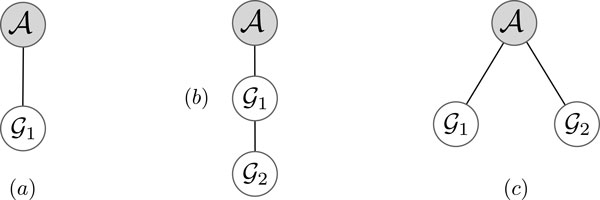
**Possible mixture trees when *k *= 1, 2**. (a) The only topology in 1-MCP. (b) Branch-tree and (c) path-tree topologies in 2-MCP.

**Figure 3:**
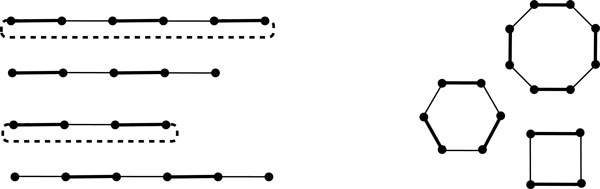
**The breakpoint graph B(G,A) with possible edges to be added to the adjacencies of  G**. Breakpoint graph B(G,A) consisting of paths and cycles. Thick edges are in A(A), and thin edges are in $A\left(G\right)$. Dashed edges are the edges that should be added to $A\left(G\right)$ for the paths whose first and last edges are in A(A).

*Proof*. In 1-MCP we have a single partial genome  G and a reference genome  A (see Figure 2-a). Since both  G and  A are matchings over the gene extremities, their breakpoint graph B(G,A) consists of some paths and cycles. Suppose *P*_1_, . . ., *P_r _*are all the paths such that the first and their last edges are adjacencies in  A. An optimal completion for  G can be obtained by adding an edge to  G which connects the end points of each *P_i_*, for 1 *≤ i ≤ r *(see Figure 3), since we only can add edges between the vertices which are not incident with any edge in $A\left(G\right)$, i.e., the end vertices of *P_i_*'s. Note that adding other possible edges just create longer paths in B(G,A). □

### 1-MCP*_c_*: circular uni-chromosomal completion

Here we consider 1-MCP*_c_*, the restricted 1-MCP for a partial genome  G that we wish to complete to a circular uni-chromosomal genome $G_{c}$. We assume that  G is not already a circular uni-chromosomal genome. Thus  G has a set F(G) of *free *extremities, i.e., the extremities that are not in any adjacency in  G. Equivalently, F(G) is the set of vertices of degree 0 in the genome graph $\text{gr}\left(G\right)$. Finding the completion $G_{c}$ corresponds to finding a partition of F(G) into pairs of extremities, i.e., into adjacencies. However, this partition cannot be arbitrary as the adjacencies defined by the partition must satisfy two constraints: (1) The resulting genome $G_{c}$ is circular uni-chromosomal, meaning that the augmented genome graph $\bar{\text{gr}}\left(G_{c}\right)$ has exactly one component, a cycle. Note that $\bar{\text{gr}}\left(G\right)$ has only path components, since $\bar{\text{gr}}\left(G\right)\subset \bar{\text{gr}}\left(G_{c}\right)$ and $G\neq G_{c}$. (2) The resulting genome $G_{c}$ must minimize the distance between the reference genome  A and $G_{c}$.

The first constraint on partitioning of F(G) is that joining extremities at ends of a same path in $\bar{\text{gr}}\left(G\right)$ by an edge, which we call an *excluded edge*, creates a cycle. This cycle must be selected carefully to obtain a uni-chromosomal genome. We define E(G) to be the set of all excluded edges.

The second constraint on partitioning of F(G) is provided by our desire to minimize the distance between the reference genome  A and $G_{c}$. For the DCJ distance, we must maximize the number $c\left(A,G_{c}\right)$ of cycles in the breakpoint graph B=B(G,A). Adding an edge to $A\left(G\right)$ increases the number of cycles in *B *if and only if the edge connects the endpoints of a same path in *B*. We call such an edge a *desired *edge and denote by $D_{A}\left(G\right)$ the set of all desired edges. Now we combine these two constraints into a graph.

**Figure 4:**
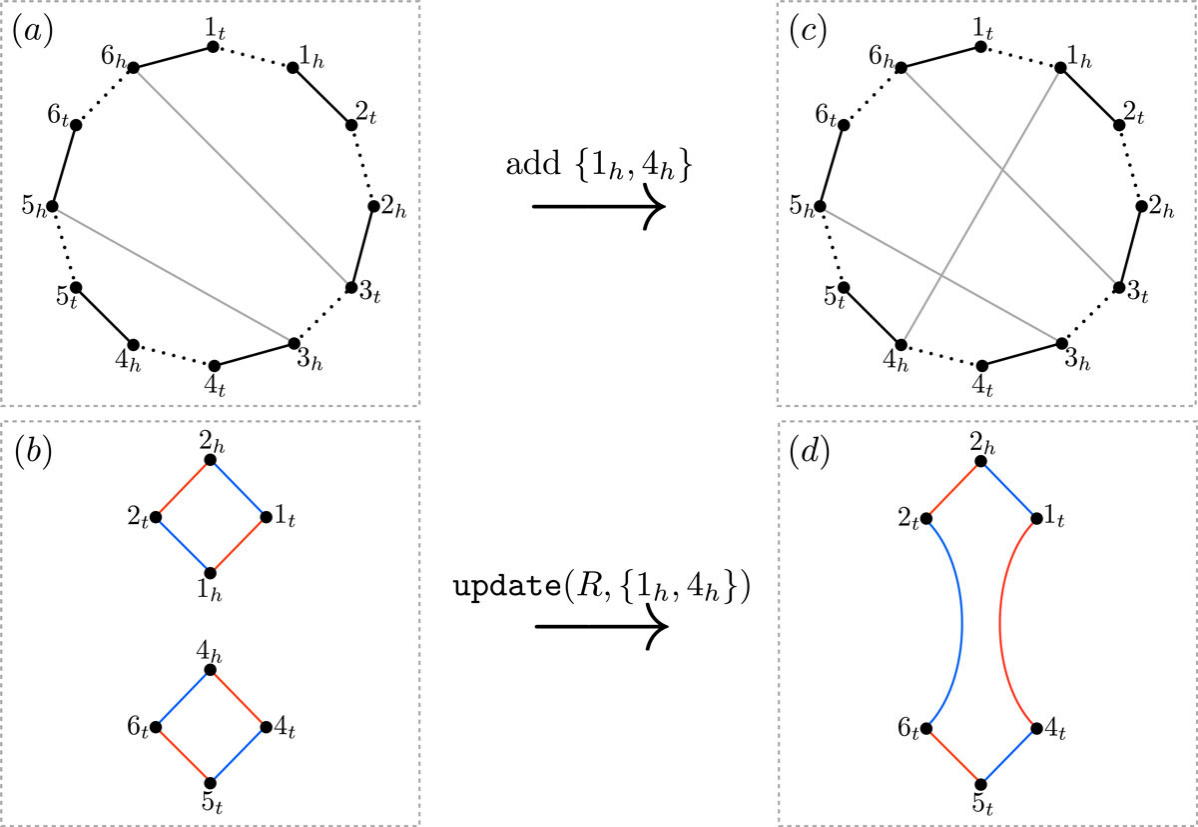
**Adding adjacencies to a partial genome  G to solve the 1-MCP*_c_***. (a) The breakpoint graph B(G,A). Gray edges indicate adjacencies of  G, black edges indicate adjacencies of  A, and the dotted edges connect extremities of the same gene. The set of free vertices is $F\left(G\right)=\left\{1_{t},1_{h},2_{t},2_{h},4_{t},4_{h},5_{t},6_{t}\right\}$. (b) The free-extremities graph R(G,A) consists of two even cycles. Blue edges are desired edges $D_{A}\left(G\right)$ and red edges are excluded edges E(G). (c) The resulting breakpoint graph after adding adjacency {1*_h_*, 4*_h_*}. (d) The resulting free-extremities graph after update(*R*, {1*_h_*, 4*_h_*}). The vertices 1*_h _*and 4*_h _*are no longer free extremities and thus are removed during update(*R*, {1*_h_*, 4*_h_*}).

We define the *free-extremities graph*, R=R(G,A) to be a bicolored graph, whose vertex set is F(G), and whose edge set is $D_{A}\left(G\right)⊔E\left(G\right)$. The edges from $D_{A}\left(G\right)$ are colored *blue *and the edges from E(G) are colored *red*. Note that *R *is a multi-graph, and *R *consists of even cycles. This is because both $D_{A}\left(G\right)$ and E(G) are perfect matchings on F(G): since both A(A) and {{*g_h_, g_t_*} *| g *is a gene in  G} are perfect matchings on the set of all gene extremities. The restriction of these perfect matchings to F(G) are $D_{A}\left(G\right)$ and E(G). See Figure 4-b. Thus, we have

(1) $$|E\left(G\right)|\;=\;|D_{A}\left(G\right)|\;=\;\frac{\left|F\left(G\right)\right|}{2}$$

To find a completion of the partial genome  G we select pairs {*u*, *v*} of free extremities from F(G) and add them as adjacencies to $A\left(G\right)$. Respecting the constraints encoded in the free-extremities graph *R*, we define a transformation update(*R*, {*u*, *v*}) that records the effect of adding adjacency {*u, v*} to  G (Figure 4). In particular, since *u *and *v *are free vertices of  G, there are paths $P_{B}^{u}$ and $P_{B}^{v}$ in *B *with an endpoint equal to *u *and *v*, respectively. Similarly, there are paths $P_{\bar{\text{gr}}\left(G\right)}^{u}$ and $P_{\bar{\text{gr}}\left(G\right)}^{v}$ in $\bar{\text{gr}}\left(G\right)$ having an endpoint equal to *u *and *v*, respectively. We may have $P_{B}^{u}=P_{B}^{v}$ or $P_{\bar{\text{gr}}\left(G\right)}^{u}=P_{\bar{\text{gr}}\left(G\right)}^{v}$. By the definition of $D_{A}\left(G\right)$, $P_{B}^{u}$ and $P_{B}^{v}$ are represented by blue edges *b^u ^*and *b^v ^*in *R *incident to *u *and *v*. Similarly by the definition of E(G), $P_{\bar{gr}\left(G\right)}^{u}$ and $P_{\bar{\text{gr}}\left(G\right)}^{v}$ are represented by red edges *r^u ^*and *r^v ^*in *R *incident to *u *and *v*. Adding the adjacency {*u*, *v*} to $A\left(G\right)$ will have the following effects on *B *and $\bar{\text{gr}}\left(G\right)$:

(i) *u *and *v *are no longer free vertices.

(ii) If $P_{B}^{u}\neq P_{B}^{v}$ then these paths merge into one path in *B *∪ {*u*, *v*}. Otherwise these paths merge to create a cycle in *B *∪{*u*, *v*}, and the number of cycles in the breakpoint graph increases by one.

(iii) If $P_{\bar{\text{gr}}\left(G\right)}^{u}\neq P_{\bar{\text{gr}}\left(G\right)}^{v}$ these paths merge into one path in $\bar{\text{gr}}\left(G\right)\cup \left\{u,v\right\}$. Otherwise these paths merge into a cycle in $\bar{\text{gr}}\left(G\right)\cup \left\{u,v\right\}$. In the latter case, we should add {*u*, *v*} as an adjacency if and only if F(G)={u,v}. This is because adding {*u*, *v*} creates a cycle component in $\bar{\text{gr}}\left(G\right)\cup \left\{u,v\right\}$ (i.e., a circular chromosome) and if there are other free vertices any subsequent completion will *not *be uni-chromosomal.

Therefore, adding the adjacency {*u*, *v*} to $A\left(G\right)$ will have three corresponding effects on *R*: removing the vertices *u *and *v *from *R *based on (i) above, identifying *b^u ^*and *b^v ^*based on (ii) above, and identifying *r^u ^*and *r^v ^*based on (iii) above. We denote this process of updating *R *by update(*R*, {*u*, *v*}). Figure 4 gives an illustration of this process.

If {*u*, *v*} is a blue edge in *R*, then update(*R*, {*u*, *v*}) increases the number of cycles in the breakpoint graph *B *by one. Hence, to find a solution to 1-MCP*_c _*we want to perform update(*R*, {*u*, *v*}) transformations with as many blue edges as possible. On the other hand, adding new adjacencies has to merge the paths in the graph $\bar{\text{gr}}\left(G\right)$ in such a way that we end with a genome with exactly one circular chromosome. Let *M_b_*(*R*) be the maximum possible number of update transformations using blue edges for the graph *R*. The following theorem provides the exact value of *M_b_*(*R*).

*Theorem *2. Suppose  G is a partial genome,  A is a reference genome, and R=R(G,A) is their free-extremities graph. We have

$$M_{b}\left(R\right)=N_{b}\left(R\right)-c\left(R\right)+1,$$

where *N_b_*(*R*) is the number of blue edges, and *c*(*R*) is the number of cycles in *R*.

*Proof*. We prove the theorem by induction on *N_b_*(*R*). Suppose *N_b_*(*R*) = 1. Then necessarily *R *consists of a cycle of length 2 with one blue and one red edge, and *c*(*R*) = 1. Thus, we update the graph *R *with the unique (and the only possible) blue edge obtaining

$$M_{b}\left(R\right)=1=N_{b}\left(R\right)-c\left(R\right)+1.$$

Now suppose *N_b_*(*R*) *>*1. Then |E(G)|>1, since $|E\left(G\right)|\;=\;|D_{A}\left(G\right)|=N_{b}$. Suppose *u*, v∈F(G), and {u,v}∉E(G), i.e., there is no red edge between *u *and *v *in *R*. Then, we have the following three cases for *u *and *v*: (i) *u *and *v *are from different cycles *C_u _*and *C_v _*in *R*, (ii) *u *and *v *are connected with a blue edge in a cycle *C *of *R*, or (iii) *u *and *v *are non-neighboring vertices in a cycle *C *of *R*.

Let *R*' = update(*R*, {*u, v*}) be the free-extremities graph after the update. Since *u *and *v *are incident with blue edges in *R*, after update(*R*, {*u*, *v*}) the number of blue edges decreases by one, i.e., *N_b_*(*R*') = *N_b_*(*R*) - 1.

Thus, by induction hypothesis

(2) $$M_{b}\left(R^{\prime }\right)=N_{b}\left(R^{\prime }\right)-c\left(R^{\prime }\right)+1=N_{b}\left(R\right)-c\left(R^{\prime }\right).$$

Considering the above cases we have:

(i) After update(*R*, {*u*, *v*}), *C_u _*and *C_v _*will shrink into one cycle, and *c*(*R*') = *c*(*R*) - 1. Thus by (2), *M_b_*(*R*') = *N_b_*(*R*) *- c*(*R*) + 1. By choosing such an edge we can update *R *with *N_b_*(*R*) *- c*(*R*) + 1 blue edges.

(ii) After update(*R*, {*u*, *v*}), *C *shrinks into a smaller cycle, and *c*(*R*') = *c*(*R*). Thus, by (2), *M_b_*(*R*') = *N_b_*(*R*) *- c*(*R*). Since {*u*, *v*} is a blue edge, we can update *R *with *N_b_*(*R*) *- c*(*R*) + 1 blue edges.

(iii) After update(*R*, {*u*, *v*}), *C *splits into two smaller cycles. Thus *c*(*R*') = *c*(*R*) + 1. Thus, by (2), *M_b_*(*R*') = *N_b_*(*R*) *- c*(*R*) *- *1. So by choosing {*u*, *v*} we can update *R *with *N_b_*(*R*) *- c*(*R*) *- *1 blue edges.

By calculations above, choosing a pair {*u*, *v*} satisfying cases (i) or (ii) will result in a greater number of update moves with blue edges, than choosing a pair satisfies the case (iii). Moreover, considering pairs {*u*, *v*} from cases (i) and (ii) gives *M_b_*(*R*) = *N_b_*(*R*) *- c*(*R*) + 1. □

We call a pair {*u*, *v*} (which may or may not be an edge in *R*) satisfying case (i) or (ii) in the proof of Theorem 2 an *optimal *adjacency. Optimal adjacencies play an important role in finding a solution of 1-MCP*_c_*: updating the free-extremities graph with these adjacencies results in the maximum number of blue edges used in update transformations. We have the following important corollary to this theorem.

*Corollary *1. Suppose  G is a partial genome and  A is a reference genome. Adding any optimal adjacency to $A\left(G\right)$ leads to a solution for 1-MCP*_c_*. In other words, for any optimal adjacency *e*, there exists a solution $G_{c}$ for 1-MCP*_c _*which includes *e *as an adjacency.

*Proof*. By Theorem 2, adding any optimal adjacency to $A\left(G\right)$ will allow the maximum number of blue edges in the update process. Since each update transformation on a blue edge increases the number of cycles in the breakpoint graph by one, a sequence of update transformations on optimal adjacencies gives a solution $G_{c}$ to 1-MCP*_c_*. Hence, if $G_{c}$ is the resulting completion of  G, we obtain the maximum number of cycles in the breakpoint graph $B\left(G_{c},A\right)$. □

A linear time (in number of genes) algorithm for solving 1-MCP*_c _*adds optimal adjacencies according to cases (i) and (ii) in Theorem 2, and is shown in Algorithm 1. The following corollary is an immediate consequence of Corollary 1 and Algorithm 1.

*Corollary *2. The 1-MCP*_c _*is solvable in linear time.

**Algorithm 1**: Solving 1-MCP*_c_*

**Input **: Partial genome  G and reference genome *A*.

**Output**: A 1-completion $G_{c}$ that is circular uni-chromosomal and maximizes $c\left(G_{c},A\right)$.

1  **begin**

2    Construct the free-extremities graph R=R(G,A);

3    $G_{c}\leftarrow G$;

4    **while ***c*(*R*) *>*1 **do**

5        *u, v ← *select two vertices from different cycles in *R*;

6        $A\left(G_{c}\right)\leftarrow A\left(G_{c}\right)\cup \left\{u,v\right\}$;

7        *R ← *update (*R, {u, v}*);

8    **while ***the number of blue edges in R >*1 **do**

9        *u, v ← *select two vertices connected via a blue edge in *R*;

10        $A\left(G_{c}\right)\leftarrow A\left(G_{c}\right)\cup \left\{u,v\right\}$;

11        *R ← *update (*R, {u, v}*);

12    Add the single remaining excluded edge in E(G) to $A\left(G_{c}\right)$;

13    Output the resulting circular uni-chromosomal genome $G_{c}$;

14  **end**

### 1**-MCP**_ℓ_**: linear uni-chromosomal completion**

In this section we consider the 1-MCP with chromosomal condition of a linear uni-chromosomal genome. We refer to this restricted problem as 1-MCP_ℓ_. We relate solutions of 1-MCP_ℓ _to solutions of 1-MCP*_c_*. Combined with the results in the previous section, we derive a linear time algorithm for 1-MCP_ℓ_.

Recall that ${ĉ}_{c}\left(G,A\right)$ is the number of alternating cycles in the breakpoint graph $B\left(G_{c},A\right)$, for any solution $G_{c}$ of 1-MCP*_c_*. Similarly, we define ${ĉ}_{\ell }\left(G,A\right)$ to be the number of alternating cycles in $B\left(G_{\ell },A\right)$, for any solution $G_{\ell }$ of 1-MCP_ℓ_. The following theorem relates the solutions of 1-MCP*_c _*to the solutions of 1-MCP_ℓ_.

*Theorem *3. Let  G be a partial genome, $A_{c}$ be a circular uni-chromosomal genome, and $A_{\ell }$ be a linear uni-chromosomal genome obtained from $A_{c}$ by removing an adjacency *e*. Suppose $A_{c}$ and $A_{\ell }$ are the reference genomes in 1-MCP*_c _*and 1-MCP_ℓ_, respectively. From any solution $G_{c}$ to 1-MCP*_c _*we obtain a solution $G_{\ell }^{\prime }$ for 1-MCP_ℓ_. Also, from any solution $G_{\ell }$ to 1-MCP_ℓ _we obtain a solution $G_{c}^{\prime }$ for 1-MCP*_c_*. Moreover, ${ĉ}_{c}\left(G,A_{c}\right)={ĉ}_{\ell }(G,A_{\ell })+\theta \left(e\right)$, where

$$\theta (e)=\{\begin{array}{ll} 2 & \text{if }e\;\text{is}\;\text{in}\;\text{a}\;\text{cycle}\;\text{in}\;B(G,A_{c}),\\ 1 & \text{otherwise}\text{.} \end{array}$$

**Figure 5:**
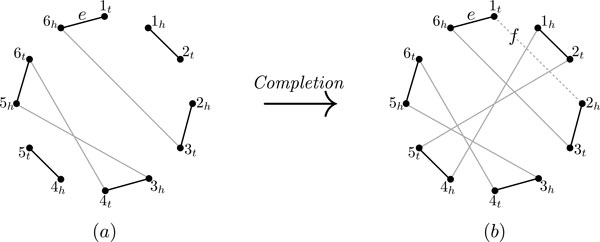
**Relating 1-MCP*_c _*and 1-MCP_ℓ_**. (a) The breakpoint graph $B=B\left(G,A_{c}\right)$; black edges are $A\left(A_{c}\right)$ and and gray edges are $A\left(G\right)$. The edge *e *= {1*_t_*, 6*_h_*} is the only edge in $A\left(A_{c}\right)\backslash A\left(A_{\ell }\right)$. Since *e *is not in a cycle component of *B*, we have *θ*(*e*) = 1. (b) The breakpoint graph $B^{\prime }=B\left(G_{c},A_{c}\right)$, where $G_{c}$ is a completion of  G and a solution to 1-MCP*_c_*. The adjacency *f *is in $A\left(G_{c}\right)$ and shown by a gray dashed edge. *B*' has two cycles, and removing *e *and *f *decreases the number of cycles by one.

*Proof*. First, suppose *e *is not in any cycle in the graph $B\left(G,A_{c}\right)$, and hence *θ*(*e*) = 1. Let $G_{c}$ be a solution to 1-MCP*_c_*, and let $G_{\ell }^{\prime }$ be a linear uni-chromosomal genome obtained from $G_{c}$ by removing an adjacency $f\in A\left(G_{c}\right)\backslash A\left(G\right)$, such that *f *and *e *are in the same cycle in $B\left(G_{c},A_{c}\right)$. Note that such edge *f *exists, since *e *is not in any cycle in $B\left(G,A_{c}\right)$ but it is in a cycle of $B\left(G_{c},A_{c}\right)$. See Figure 5. Both gr$\left(G_{c}\right)$ and $\text{gr}\left(A_{c}\right)$ are perfect matchings as $A_{c}$ and $G_{c}$ are both circular. Removing the edges *e *and *f *from $B\left(G_{c},A_{c}\right)$ will decrease the number of cycles by exactly one since *e *and *f *are in a same cycle in $B\left(G_{c},A_{c}\right)$. Hence $c\left(G_{\ell }^{\prime },A_{\ell }\right)=c\left(G_{c},A_{c}\right)-1$, and we have,

(3) $${ĉ}_{c}\left(G,A_{c}\right)-1=c\left(G_{\ell }^{\prime },A_{\ell }\right)\leq {ĉ}_{\ell }(G,A_{\ell }),$$

where the last inequality follows from the definition of ${ĉ}_{\ell }\left(G,A_{\ell }\right)$ as the largest number of cycles in *any *linear chromosomal completion of  G.

Now suppose $G_{\ell }$ is a solution to 1-MCP_ℓ_, so $|E\left(G_{\ell }\right)|=|E\left(A_{\ell }\right)|=1$. Assume $E\left(G_{\ell }\right)=\left\{f^{\prime }\right\}$. Let $G_{c}^{\prime }$ be the circular uni-chromosomal genome obtained by adding $f^{\prime }$ to $G_{\ell }$. Note that there is at least one path component in $B\left(G_{\ell },A_{\ell }\right)$ which becomes a cycle after adding the edges *f*' to $A\left(G_{\ell }\right)$ and *e *to $A\left(A_{\ell }\right)$. Hence, ${ĉ}_{\ell }\left(G,A_{\ell }\right)+1=c\left(G_{\ell },A_{\ell }\right)+1\leq c\left(G_{c}^{\prime },A_{c}\right)\leq {ĉ}_{c}\left(G,A_{}\right)$, and we have

(4) $${ĉ}_{\ell }\left(G_{\ell },A_{\ell }\right)\leq {ĉ}_{c}\left(G,A_{c}\right)-1.$$

Thus by (3) and (4) we have ${ĉ}_{c}\left(G,A_{c}\right)={ĉ}_{\ell }\left(G,A_{\ell }\right)+1$, which implies that $c\left(G_{c}^{\prime },A_{c}\right)={ĉ}_{c}\left(G,A_{c}\right)$ and $c\left(G_{\ell }^{\prime },A_{\ell }\right)={ĉ}_{\ell }\left(G,A_{\ell }\right)$. This means that $G_{c}^{\prime }$ and $G_{\ell }^{\prime }$ are solutions to 1-MCP*_c _*and 1-MCP_ℓ _that are obtained from $G_{\ell }$ and $G_{c}$, respectively, which completes the proof for the case *θ*(*e*) = 1.

Now suppose *e *is in a cycle in $B\left(G,A_{c}\right)$, and thus *θ*(*e*) = 2. Using the same argument above, we have ${ĉ}_{c}\left(G,A_{c}\right)-2=c\left(G_{\ell }^{\prime },A_{\ell }\right)\leq {ĉ}_{\ell }\left(G,A_{\ell }\right)$ since we cannot find such edge *f *and the number of cycles in $B\left(G_{c},A_{c}\right)$ decreases by two, when we remove an edge from $G_{c}$ (to obtain a linear genome), and *e *from $A_{c}$ (to obtain the genome $A_{\ell }$). Also, ${ĉ}_{\ell }\left(G,A_{\ell }\right)+2\leq {ĉ}_{c}\left(G,A_{c}\right)$, as adding the excluded edges of $A_{\ell }$ and $G_{\ell }$ will increase the number of cycles by 2. Thus, for this case we have ${ĉ}_{c}\left(G,A\right)={ĉ}_{\ell }\left(G,A\right)+2$ □

Notice that the function *θ *depends only on the partial genome  G and the reference genome $A_{c}$, and not on the completion $G_{c}$. Also, it is easy to see that *θ *is computable in linear time (in number of genes). We have the following corollary.

*Corollary *3. The 1-MCP_ℓ _is solvable in linear time.

*Proof*. Suppose  G is a partial genome and $A_{\ell }$ is a linear chromosomal reference genome. Since $A_{\ell }$ is linear and uni-chromosomal, $|E\left(A_{\ell }\right)|=1$. Assume that $E\left(A_{\ell }\right)=\left\{e\right\}$. Let $A_{c}$ be the circular uni-chromosomal genome obtained by adding *e *to $A\left(A_{\ell }\right)$. Using Algorithm 1 we obtain a solution $G_{c}$ for 1-MCP*_c _*with $A_{c}$ as the reference genome. Then by Theorem 3, we can transform the solution $G_{c}$ to a linear uni-chromosomal completion $G_{\ell }$ in linear time in the following way: If there exists an edge $f\in A\left(G_{c}\right)\backslash A\left(G\right)$ such that *f *and *e *are in the same cycle of the breakpoint graph $B\left(G_{c},A_{c}\right)$, i.e. *θ*(*e*) = 1, remove *f *from $A\left(G_{c}\right)$. Otherwise *θ*(*e*) = 2 and we remove an arbitrary edge from $A\left(G_{c}\right)$ to make a linear uni-chromosomal genome. Therefore, we obtain a solution to 1-MCP_ℓ _by viewing  G as a partial genome for a 1-MCP*_c_*, solving the problem, and converting the solution $G_{c}$ of 1-MCP*_c _*into a solution $G_{\ell }$ for 1-MCP_ℓ_. Since all of these steps are done in linear time (in number of genes), the proof is complete. □

### (3 *≤ k*)-MCP

In the unrestricted case of the *k*-MCP, the completion of a partial genome is always possible as we can add adjacencies and telomeres arbitrarily to the partial genome, since there is no restriction on the number and type of chromosomes in the resulting genome. The hardness of showing the existence of a *k*-completion derives from the fact that finding a *k*-completion for the partial multi-genome results in a proper edge coloring for the genome graph of the partial multi-genome.

Let *G *= (*V*, *E*) be a graph. We define the *edge-chromatic number *of *G*, denoted *χ'*(*G*), to be the minimum number of colors required to obtain an edge-coloring of *G*. For each edge-coloring of *G *a *color class *is a set of all edges with a specific color. A color class defines a matching in the graph since no two edges of the same color share a vertex.

The following proposition shows the relation between the edge-coloring of a genome graph and the edge color classes of the corresponding breakpoint graph.

*Proposition *1. If  M is a multi-genome of *k *genomes then $\chi^{\prime }\left(\text{gr}\left(M\right)\right)\leq k$.

*Proof*. Suppose  M is a mixture of *k *genomes $G_{1},\ldots G_{k}$. Then the breakpoint graph $B=B\left(G_{1},\ldots ,G_{k}\right)$ can be partitioned into the sets $A\left(G_{i}\right)$ of adjacencies, and each $A\left(G_{i}\right)$ can be considered as color class. So the edges of *B *can be colored with *k *colors. Since *B *and gr(M) are isomorphic, we have $\chi^{\prime }\left(\text{gr}\left(M\right)\right)\leq k$. □

Using the same argument as in Proposition 1 we have:

*Lemma *1. If  M is a partial multi-genome of *k *partial genomes then $\chi^{\prime }\left(\text{gr}\left(M\right)\right)\leq k$.

Now, in the following theorem we show a relation between the edge-coloring of a genome graph and the *k*-completion of the corresponding partial multi-genomes.

*Theorem *4. Let  M be a partial multi-genome. Then  M has an unrestricted *k*-completion if and only if $\chi^{\prime }\left(\text{gr}\left(M\right)\right)\leq k$, for any positive integer *k*.

*Proof*. (⇒) If  M has a *k *completion, then it can be considered as a partial multi-genome of *k *genomes. Then by Lemma 1 we have $\chi^{\prime }\left(\text{gr}\left(M\right)\right)\leq k$.

(⇐) Now assume that $\chi^{\prime }\left(\text{gr}\left(M\right)\right)\leq k$. Hence, we can color the edges of gr(M) with *k *colors. If *C*_1_, . . ., *C_k _*are the color classes of *G*, we have ${⊔}_{i=1}^{k}C_{i}=E\left(G\right)$. Each *C_i _*is a matching in the graph gr(M), and is a set of adjacencies among the gene extremities. So we can define a partial genome $G_{i}^{\prime }$ by adjacencies $A\left(G_{i}^{\prime }\right)=C_{i}$. The color classes partition the edges of gr(M) into *k *matchings, and we have $M={⊔}_{i=1}^{k}G_{i}^{\prime }$. Since there is no restriction on the completions, taking any completion $G_{i}$ for each $G_{i}^{\prime }$ results in a a *k*-completion $M^{k}=\left\{G_{1},\ldots G_{k}\right\}$ for  M; because $M={⊔}_{i=1}^{k}G_{i}^{\prime }\subseteq \;{⊔}_{i=1}^{k}G_{i}$. □

Now, by Theorem 4 and using the following two classic theorems, we show that deciding whether there exists a valid solution to a (*k ≥ *3)-MCP is NP-complete. For a graph *G *let Δ(*G*) be the maximum degree of *G*.

*Theorem *5 (Vizing [14]). If *G *is a simple graph, *χ'*(*G*) = Δ(*G*) or Δ(*G*) + 1.

*Theorem *6 (Holyler [15]). For a graph *G*, deciding whether *χ'*(*G*) = Δ(*G*) or Δ(*G*) + 1 is NP-complete, if Δ(*G*) *≥ *3.

*Corollary *4. If *k ≥ *3, deciding whether there exists a valid solution to the unrestricted *k*-MCP is NP-complete.

*Proof*. In order to prove this corollary we reduce the edge-coloring problem to *k*-MCP. Suppose *G *= (*V*, *E*) is a simple graph and *k *= Δ(*G*) *≥ *3. If *|V | *is not even, add an isolated vertex so that the number of vertices in *G *is 2*n *for some positive integer *n*. Consider these 2*n *vertices as gene extremities of a set of *n *genes. Now, *G *defines a partial multi-genome  M on these *n *genes, since the *k*-MCP is unrestricted and *any *graph can be considered as a partial multi-genome with no restriction on the chromosomal structure of its partial genomes. If there is a polynomial algorithm for *k*-MCP, we can input to this algorithm  M as the partial multi-genome, along with an arbitrary distance function *ϕ *and a healthy reference  A. First, suppose the algorithm gives a valid output. Since the algorithm is polynomial, we can find a *k*-completion for  M in polynomial time, and by Theorem 4, we can find an edge coloring of *G *with *k *colors in polynomial time.This implies that the *χ'*(*G*) *≤ k*. Now if the algorithm does not give a valid output, by Theorem 4 we have *χ'*(*G*) *> k*. This implies that the *k*-MCP is NP-complete, since the genome graph of a partial multi-genome is always a multigraph and the class of simple graphs is a subset of the class of multigraphs. □

Note that in Corollary 4 we only considered the unrestricted version of *k*-MCP. This allows us to assume that for each (multi-) graph *G *there exists a partial multi-genome  M such that *G *and gr(M) are isomorphic.Thus, if $\bar{M}=\{\text{gr}\left(M^{\prime }\right)$ | for all partial multi-genomes $M^{\prime }$} and if  Ḡ is the set of all multi-graphs, then $\bar{M}=Ḡ$. However, one can restrict the *k*-MCP by taking a set of chromosomal conditions. Consequently we may have $\bar{M}⊊Ḡ$ such that the new restricted k-MCP is polynomially tractable for all partial multi-genomes (whose genome graph is in $\bar{M}$).

*Corollary *5. If *k *≥ 3, then the unrestricted *k*-MCP is NP-complete.

*Proof*. Since in solving a *k*-MCP we need to find a *k*-completion for its partial multi-genome, by Corollary 4 the proof is complete. □

### 2**-MCP**

In this section, we prove that the unrestricted 2-MCP, and the restricted 2-MCP where all chromosomes are circular (i.e., C={circular}), are NP-complete for DCJ distance. The NP-completeness of the unrestricted 2-MCP is done by a reduction from *MAX 3-AND problem*. The MAX 3-AND is a satisfiability problem, where given a set of conjunctions, each with 3 literals, the goal is to determine an assignment of Boolean value to each variable that maximizes the number of satisfied conjunctions. Note that in 2-MCP there are only two possible topologies for the mixture tree: the *branch-tree *and *path-tree *(Figure 2-b, c).

*Theorem *7. The unrestricted 2-MCP with DCJ distance is NP-complete.

In order to provide the proof of this theorem, we need the following lemmas.

*Lemma *2. Suppose  M is a partial multi-genome whose genome graph, gr(M), consists of *m *cycles *C*_1_, . . ., *C_m _*with even lengths, and  A is a reference genome which consists of ℓ edges (i.e., it has ℓ adjacencies). Assume that there are ℓ' cycles among the cycles in gr(M) such that no edge in *A *is connected to any of their vertices. If ℓ' *>*2ℓ then in every solution to the 2-MCP, the optimal mixture tree is a path-tree.

*Proof*. Note that in 2-MCP there are only two possible topologies for the mixture tree: the *branch-tree *and *path-tree *(Figure 2-b, c). Since the degree of each vertex in gr(M) is two, if we partition the edges of gr(M) into two perfect matchings $G_{1}^{\prime }$ and $G_{2}^{\prime }$. Therefore, for any 2-completion $M^{2}=\left\{G_{1},G_{2}\right\}$ we have $G_{1}^{\prime }=G_{1}$ and $G_{2}^{\prime }=G_{2}$, since *G*_1 _and *G*_2 _are maximal (and circular) and we cannot add any edge to them. Also, for each $G_{i}\left(i=1,2\right)$ we have $G_{i}=\cup_{j=1}^{m}M_{ij}$, where *M_ij _*is a perfect matching on vertices of *C_j_*. Obviously, $c\left(G_{1},G_{2}\right)=m$. We have $c\left(A,G_{i}\right)\leq \ell$ for *i *= 1, 2, since  A has ℓ edges and each of them can be in at most one cycle in $B\left(G_{i},A\right)$. Therefore,

$$\begin{array}{ccc} c\left(A,G_{\text{1}}\right)+c\left(G_{\text{1}},G_{\text{2}}\right) & \geq c\left(G_{\text{1}},G_{\text{2}}\right)=m\geq \ell^{\prime }>\text{2}\ell & \\ & \geq c\left(A,G_{\text{1}}\right)+c\left(A,G_{\text{2}}\right), \end{array}$$

which shows that the *d_DCJ_*-value of a path tree is smaller than the *d_DCJ _*-value of a branch tree, and completes the proof. □

*Lemma *3. Any MAX 3-SAT instance is reducible to a MAX 3-AND instance. Moreover, MAX 3-AND is NP-complete.

*Proof*. Let Δ = ℓ_1 _V ℓ_2 _V ℓ_3 _be a clause (disjunction) of three literals. Define

$$L\left(\text{Δ}\right)=\left\{\left(t_{\text{1}}\wedge t_{\text{2}}\wedge t_{\text{3}}\right)|1\leq i\leq 3,t_{i}\in \left\{\ell_{i},{\bar{\ell }}_{i}\right\},\left(t_{1},t_{2},t_{3}\right)\neq \left({\bar{\ell }}_{1},{\bar{\ell }}_{2},{\bar{\ell }}_{3}\right)\right\}.$$

By using basic Boolean rules we have Δ ⇔ V_*S*∈ℓ(Δ) _*S*.

Now, suppose  I is a MAX 3-SAT instance which has *m *clauses Δ_1_, . . ., Δ*_m. _*Let $I^{\prime }$ be an instance of MAX 3-AND which consists of all the conjunctions in $\cup_{j=1}^{m}L\left(\Delta_{j}\right).$ Since for every assignment to the variables at most one conjunction in *L*(Δ*_j_*), 1 *≤ j ≤ m*, is satisfied and this happens if and only if Δ*_j _*is satisfied, then every optimal assignment to the variables in  I will be also an optimal assignment to the variables in  I. Therefore, MAX 3-SAT is reducible to MAX 3-AND, which implies that MAX 3-AND is NP-complete, as MAX 3-SAT is NP-complete [16]. □

**Figure 6:**
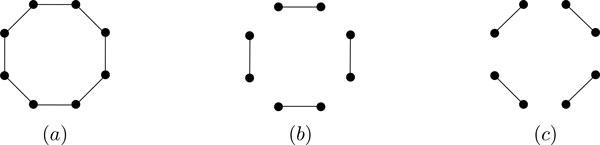
**Representing variables with cycles**. (a) A variable represented by a cycle, (b) a true matching, and (b) a false matching.

**Figure 7:**
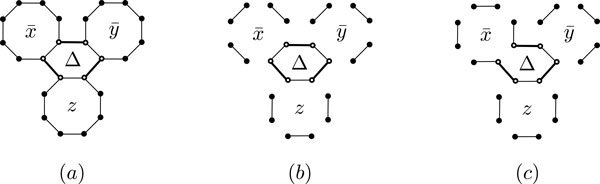
**Representing conjunctions with cycles**. (a) Three cycles representing the literals $\bar{x}$,  ȳ, and *z*, and the conjunction edges (bold) for a conjunction $\text{Δ}=\bar{x}\wedge ȳ\wedge z$. (b) For *x *= *y *= false and *z *= true we obtain the conjunction-cycle Δ of length 6. (c) Any other assignment (e.g., *x *= true) destroys the conjunction cycle.

Now, consider an instance  I of the MAX 3-AND problem. We show how to represent  I by a genome graph and a reference genome, to make a reduction from MAX 3-AND to 2-MCP. Suppose we represent a variable *x *with a cycle *C *of even length, which we will call a *variable-cycle *(see Figure 6-a). This cycle has exactly two perfect matchings. We label one of these the *true *matching, *T*(*x*), and the other one the *false *matching, *F*(*x*) (see Figure 6-b, c). We represent an assignment to a variable by choosing one of the matchings *T*(*x*) and *F*(*x*) and **remove **the edges in the other matching (see Figure 7).

Let ℓ(*x*_1_), ℓ(*x*_2_), ℓ(*x*_3_) be three literals of variables *x*_1_, *x*_2_, *x*_3_, and Δ = (ℓ(*x*_1_) Λ ℓ(*x*_2_) Λ ℓ(*x*_3_)) be a conjunction in  I. A *conjunction-cycle *of Δ is a cycle which is obtained as follows:

1. For each *i *∈ {1, 2, 3} consider an edge in *T*(*x_i_*) if ℓ(*x_i_*) = *x_i_*. If $\ell \left(x_{i}\right)={\bar{x}}_{i}$ take an edge in *F*(*x_i_*).

2. Add three new edges, called *conjunction-edges*, to the three edges we chose in the previous step, and build a cycle of length 6. This cycle is a conjunction-cycle of Δ.

It is easy to see that an assignment *α *to *x_i_*'s satisfy the conjunction Δ if and only if the corresponding matching assignment to *α *keeps all the edges in the conjunction-cycle of Δ. We call a representation of a MAX 3-AND instance  I with cycles and conjunction-cycles *a graphical representation of * I.

If the literals of a variable appear in at most *t *conjunctions, and the variable-cycles have length at least 4*t*, then by choosing the edges of conjunction-cycles properly, we have a graphical representation of a MAX 3-AND instance, where no edge in a variable-cycle is incident with two conjunction edges from different conjunction-cycles. This implies the following lemma:

*Lemma *4. For each MAX 3-AND instance  I there exists a graphical representation $I_{g}$ such that any as-signments to the variables in  I which maximizes the number of satisfied conjunctions, induces a matching assignment that maximizes the number of conjunction-cycles, and vice versa.

Combining Lemmas 2-4 gives the proof of Theorem 7.

*Proof of Theorem 7*. Since the MAX 3-AND is NP-complete by Lemma 3, it suffices to reduce the MAX 3-AND problem to the 2-MCP. Suppose  I is a MAX 3-AND instance. Assume  I has *m *conjunctions. We can add 3*m *+ 1 new conjunctions *δ*_1_, . . ., *δ*_3*m*+1 _where each *δ_i _*consists of a new single variable *x_δi_*; obviously in any optimal assignment the value of all the *x_δi_*'s should be true. Now by Lemma 4, there is a graphical representation $I_{g}$ such that finding an optimal assignment in  I is equivalent to finding a matching for each variable-cycle such that the number of preserved conjunction-cycles are maximized. Note that there are 3*m *conjunction-edges and 3*m *+ 1 variable-cycles which are not connected to any conjunction-edge. Now, consider all the vertices in $I_{g}$ as gene extremities, and all the edges in the variable-cycles as the adjacencies of a partial multi-genome *G*. Also, consider all the conjunction-edges as the adjacencies of a reference healthy genome  A. In the 2-MCP problem with partial multi-genome *G *and reference healthy genome  A, the optimal tree is forced to be a path-tree by Lemma 2 (Figure 2). Therefore, in the optimal solution of this 2-MCP, $G_{1}$ should be a genome such that the number of cycles in the breakpoint graph $B\left(G_{1},A\right)$ is maximized, i.e., the number of conjunction-cycles are maximized. Since $G_{1}$ is a union of perfect matchings of the variable-cycles (see the proof of Lemma 2) it induces an assignment for the variables which maximizes the number satisfied conjunctions, and this completes the proof. □

We end this section by considering the restricted version of *k*-MCP, where the chromosomal condition set is {circular}, i.e. all genomes have all circular chromosomes. We denote this restricted version by *k*-MCP*_c_*, and the unrestricted version of *k*-MCP by *k*-MCP_∅_. If opt(*k*-MCP*_c_*) and opt(*k*-MCP_∅_) are the *d_DCJ_*-value of a solution to *k*-MCP*_c _*and *k*-MCP_∅_, respectively, then:

*Theorem *8. For the *k*-MCP*_c _*and *k*-MCP_∅ _versions of *k*-MCP with DCJ distance we have

$$\text{opt}\left(k–\text{MC}{\text{P}}_{c}\right)=\text{opt}\left(k–\text{MC}{\text{P}}_{\emptyset }\right).$$

*Proof*. First note that each solution to *k*-MCP*_c _*is also a solution of *k*-MCP_∅_, since there is no restriction in *k*-MCP. Hence, opt(*k*-MCP*_c_*) *≥ *opt(*k*-MCP_∅_). Second, for each solution to *k*-MCP_∅ _if the resulting genomes are not circular we can add new edges to the genomes and make them circular. By adding the new edges the number of cycles in the breakpoint graph does not decrease which implies that the *d_DCJ_*-value of the newly obtained genomes is not larger than opt(*k*-MCP_∅_). Therefore, these circular genomes form a solution of *k*-MCP_∅_. So opt(*k*-MCP*_c_*) *≤ *opt(*k*-MCP_∅_) completing the proof. □

Combining this theorem and Theorem 7 we have

*Corollary *6. If *k ≥ *2, then *k*-MCP*_c _*with DCJ distance is NP-complete.

## Discussion and conclusion

In this paper we introduced the *k*-Minimum Completion Problem (*k*-MCP) motivated by the type of data produced in current cancer genome sequencing studies. We showed the following results. (1) A linear time algorithm for the unrestricted 1-MCP; (2) a linear time algorithm for the restricted versions 1-MCP where the genomes are circular or linear; i.e. the chromosomal condition set  C is {circular, uni-chromosomal} or  C is {linear, uni-chromosomal}; (3) the unrestricted *k*-MCP is NP-complete for any distance when *k ≥ *3; and (4) the 2-MCP with DCJ distance is NP-complete in the unrestricted version and with the condition that all chromosomes are circular, i.e. C={circular}. These results lay the foundation for future algorithmic studies of the *k*-MCP and the application of these algorithms to real cancer sequencing data.

There are numerous further directions to pursue. As noted in the introduction, the model described in this paper does not consider all the complexities of cancer genome sequencing: most importantly copy number aberrations (duplications and deletions) and errors in the measured adjacencies are important features of cancer genome sequencing and should be addressed.

To handle errors, one might consider weighted versions of the *k*-MCP where adjacencies have a weight corresponding to the confidence in the measurement. Regarding the current model, further work is needed on different chromosomal conditions, genomic distances, or other constraints on the relationships between the genomes in the mixture. For example, the case of linear chromosomes demands further attention, as human chromosomes are linear, although circular chromosomes have been observed in cancer [17]. Similarly, one may impose an upper bound on the number of chromosomes. One may also place restrictions on the structure of the mixture tree.

Another direction is to derive approximation algorithms. In the *k*-MCP we aim to minimize distance over all possible *k*-completion and mixture trees simultaneously. However, by separating the completion and distance optimization steps, one may employ techniques that have developed for other problems. For example, one may try to first complete the partial multi-genomes using some clustering techniques, as have been employed in metagenomic studies [18]. With complete genomes, one could then try to find optimal mixture trees rooted at the reference genome. Depending on the allowed structure of the mixture tree, techniques from genome rearrangement phylogeny problems may be employed. For example, in the case of 2-MCP, if the complete genomes are the leaves of the mixture tree, then the problem becomes the *median problem *(with the reference genome genome as the third genome) [5,13]. Alternatively, if the genomes are the vertices of the mixture tree, then the tree construction problem becomes the problem of finding a minimum spanning tree, which is in generally easier. In between these extremes, where some of the genomes in the mixture are the leaves and some are intermediate nodes (ancestors), the problem becomes a Steiner tree problem. In the cancer application, any of these cases might provide useful approximations, as the process of clonal evolution of cancer [1] might mean that cells at intermediate stages of cancer progression remain present in the tumor.

## Competing interests

The authors declare that they have no competing interests.

## Authors' contributions

All authors contributed equally to this work.

## References

[B1] Nowell PC The clonal evolution of tumor cell populations Science 1976 194 4260 23 28 10.1126/science.959840 959840

[B2] Raphael BJ Volik S Collins C Pevzner PA Reconstructing tumor genome architectures Bioinformatics 2003 19 Suppl 2 i162 171 10.1093/bioinformatics/btg1074 14534186

[B3] Meyerson M Gabriel S Getz G Advances in understanding cancer genomes through second-generation sequencing Nat Rev Genet 2010 11 10 685 696 10.1038/nrg2841 20847746

[B4] Yancopoulos S Attie O Friedberg R Efficient sorting of genomic permutations by translocation, in-version and block interchange Bioinformatics 2005 21 16 3340 3346 10.1093/bioinformatics/bti535 15951307

[B5] Tannier E Zheng C Sankoff D Multichromosomal median and halving problems under different genomic distances BMC Bioinformatics 2009 10 10.1186/1471-2105-10-120 PMC2683817 19386099

[B6] Hannenhalli S Pevzner PA Transforming Men into Mice (Polynomial Algorithm for Genomic Distance Problem) FOCS 1995 IEEE Computer Society 581 592

[B7] Hannenhalli S Pevzner PA Transforming Cabbage into Turnip: Polynomial Algorithm for Sorting Signed Permutations by Reversals J ACM 1999 46 1 27 10.1145/300515.300516

[B8] Bergeron A Mixtacki J Stoye J A new linear time algorithm to compute the genomic distance via the double cut and join distance Theor Comput Sci 2009 410 51 5300 5316 10.1016/j.tcs.2009.09.008

[B9] El-Mabrouk N Sankoff D The Reconstruction of Doubled Genomes SIAM J Comput 2003 32 3 754 792 10.1137/S0097539700377177

[B10] Warren R Sankoff D Tannier E Genome Aliquoting Revisited RECOMB-CG, Volume 6398 of Lecture Notes in Computer Science 2010 Springer 1 12

[B11] Zheng C Lenert A Sankoff D Reversal distance for partially ordered genomes ISMB (Supplement of Bioinformatics) 2005 502 508 10.1093/bioinformatics/bti1037 15961497

[B12] Gaul É Blanchette M Bourque G, El-Mabrouk N Ordering Partially Assembled Genomes Using Gene Arrangements Comparative Genomics, Volume 4205 of Lecture Notes in Computer Science 2006 Springer 113 128

[B13] Xu AW Nelson CE, Vialette S A Fast and Exact Algorithm for the Median of Three Problem-A Graph Decomposition Approach RECOMB-CG, Volume 5267 of Lecture Notes in Computer Science 2008 Springer 184 197

[B14] Vizing VG On an estimate of the chromatic class of a *p*-graph. (Russian) Diskret Analiz 1964 3 25 30

[B15] Holyer I The NP-Completeness of Edge-Coloring SIAM J Comput 1981 10 4 718 720 10.1137/0210055

[B16] Cook SA Harrison MA, Banerji RB, Ullman JD The Complexity of Theorem-Proving Procedures STOC 1971 ACM 151 158

[B17] Raphael BJ Pevzner PA Reconstructing tumor amplisomes ISMB/ECCB (Supplement of Bioinformat-ics) 2004 265 273 10.1093/bioinformatics/bth931 15262808

[B18] Wu YW Ye Y Berger B A Novel Abundance-Based Algorithm for Binning Metagenomic Sequences Using ℓ-Tuples RECOMB, Volume 6044 of Lecture Notes in Computer Science 2010 Springer 535 549

